# Exogenous Spermidine Improves Seed Germination of White Clover under Water Stress via Involvement in Starch Metabolism, Antioxidant Defenses and Relevant Gene Expression

**DOI:** 10.3390/molecules191118003

**Published:** 2014-11-05

**Authors:** Zhou Li, Yan Peng, Xin-Quan Zhang, Xiao Ma, Lin-Kai Huang, Yan-Hong Yan

**Affiliations:** College of Animal Science and Technology, Sichuan Agricultural University, Ya’an 625014, China; E-Mails: lizhou1986814@163.com (Z.L.); zhangxq@sicau.edu.cn (X.-Q.Z.); maroar@126.com (X.M.); huanglinkai@sicau.edu.cn (L.-K.H.); yanyanhong3588284@126.com (Y.-H.Y.)

**Keywords:** amylase, drought, gene expression, reactive oxygen species, white clover (*Trifolium repens* L.)

## Abstract

This study was designed to determine the effect of exogenous spermidine (Spd) (30 μM) on white clover seed germination under water stress induced by polyethylene glycol 6000. Use of seed priming with Spd improved seed germination percentage, germination vigor, germination index, root viability and length, and shortened mean germination time under different water stress conditions. Seedling fresh weight and dry weight also increased significantly in Spd-treated seeds compared with control (seeds primed with distilled water). Improved starch metabolism was considered a possible reason for this seed invigoration, since seeds primed with Spd had significantly increased α-amylase/β-amylase activities, reducing sugar, fructose and glucose content and transcript level of β-amylase gene but not transcript level of α-amylase gene. In addition, the physiological effects of exogenous Spd on improving seeds’ tolerance to water deficit during germination were reflected by lower lipid peroxidation levels, better cell membrane stability and significant higher seed vigour index in seedlings. Enhanced antioxidant enzyme activities (superoxide dismutase, peroxidase, catalase and ascorbate peroxidase), ascorbate-glutathione cycle (ASC-GSH cycle) and transcript level of genes encoding antioxidant enzymes induced by exogenous Spd may be one of the critical reasons behind acquired drought tolerance through scavenging of reactive oxygen species (ROS) in water-stressed white clover seeds. The results indicate that Spd plays an important function as a stress-protective compound or physiological activator.

## 1. Introduction

Drought is a severe challenge to agricultural production worldwide, and water is one of the necessary conditions for seed germination. Drought greatly affects seed germination, and consequently induces a reduction in germination rate and a delay in the initiation of the germination and seedling establishment [1,2]. In many crop plants, including white clover (*Trifolium repens* L.), seed germination and early seedling growth are the most sensitive stages to water deficit [3,4]. Therefore, it is worthwhile to investigate the molecular and physiological mechanisms of poor germination induced by water deficit and to establish suitable measures which might alleviate the negative effects of seed germination caused by water deficit. Seed priming is a technique by which seeds are partially hydrated to a point where germination-related metabolic processes begin but radicle emergence does not occur [5]. Primed seeds usually show improved germination percentages [6], and many physiological, biochemical and molecular changes have been found as the possible basis of this improved performance [7,8].

Seed germination is the strongest period of life activity in all life periods of a plant and is also the basis of plant formation. During this period, the metabolism of fats, proteins and carbohydrates provides substances and energy for seedling growth [9]. Starch can be degraded by hydrolysis in reactions catalyzed by the amylase enzyme family to supply carbon sources [10,11]. The α-amylases are endoenzymes which can produce shorter chains called limit dextrins, glucose, and maltose by breaking α-(1, 4) bonds at random within polysaccharide chains. The β-amylases are exohydrolases that hydrolyze α-(1, 4) bonds in polysaccharides to remove successive maltose units from the non-reducing ends of α-1, 4-linked poly- and oligoglucans until the first α-1, 6-branching point along the substrate molecule is encountered [12]. Soluble sugars and amylase activities have been positively correlated with seedling vigor [7]. Exogenous nitric oxide improves seeds germination and seedling establishment in wheat involved an increase of amylase and starch metabolism under abiotic stress [13]. Furthermore, it has been demonstrated that drought stress could cause an imbalance between the generation and quenching of reactive oxygen species (ROS). ROS, such as superoxide radicals (O_2_^−^), hydrogen peroxide (H_2_O_2_) and hydroxyl radicals (^−^OH), which are highly reactive in the absence of effective protective mechanism, can seriously damage plants by lipid peroxidation, protein degradation and cell death [14,15], and therefore the most important factors responsible for stress tolerance in plants should be their antioxidant defenses, including enzymatic and nonenzymatic constituents [16].

Polyamines (PAs), including putrescine (Put), spermidine (Spd) and spermine (Spm), are ubiquitous low-molecular-weight aliphatic amines involved in various biochemical and physiological processes related to the regulation of plant growth and development [17,18]. More and more evidence has proved that PAs take part in the regulation of plants’ responses to various environmental stresses like drought or osmotic stress, salinity, heat and chilling by directly binding to membrane phospholipids, directly scavenging free radicals, osmotic adjustment, maintaining a cation-anion balance and binding to the antioxidant enzymes to increase their activities [19,20,21]. The fact that PAs have been shown to activate protein synthesis suggests that they are activators of this process early in germination [22]. Seed priming with PAs solutions could improve germination and stress tolerance of seedlings under abiotic stress. The study of Xu *et al.* [23] showed that Put priming treatment could improve germination percentage and enhance the chilling tolerance of tobacco seedlings. Glucose-induced inhibition of seed germination in *Lotus japonicus* can be alleviated by Spm [24]. Similarly, seed priming with Spd has been found effective to improve the germination and early seedling growth in sunflower [25] and rice [26]. Exogenous Spd also helped to maintain antioxidant enzyme activities of Welsh onion (*Allium fistulosum*) which are able to moderate the radical scavenging system and lessen in this way the oxidative stress [27]. Moreover, overexpression of Spd synthase gene in transgenic *Arabidopsis thaliana* maintained higher levels of Spd content and enhanced tolerance to chilling, salinity, hyperosmosis and drought relative to the wild-type plants, which strongly suggests that Spd plays an important role as a signaling regulator in stress signaling pathways, leading to build-up of stress tolerance mechanisms in plants under stress conditions [28]. These studies further highlight the importance of Spd for stress tolerance in plants.

Up to now, most previous studies investigating the improvement of germination and drought tolerance by seed priming with Spd only studied antioxidant enzyme activities and carbohydrates. Limited research has focused on the gene expression patterns in conjunction with the underlying enzymes promoting drought tolerance during seed germination, and no report is available concerning starch metabolism and the effects of amylase genes. The role of Spd in regulation of root viability during seeds germination under water stress is still unclear and not fully understood. Further understanding the association of enzyme activity and gene expression under water deficit is important for studying molecular factors controlling antioxidant defense and starch metabolism. Consequently, the objectives of this study were: (i) to test the effects of seed priming with Spd on the germination characteristics of white clover under water deficit conditions; (ii) to determine whether the acquired water stress tolerance induced by exogenous Spd is associated with changes in the antioxidant defense system and starch metabolism; and (iii) to assess the relationship between enzyme activity and gene expression during seed germination under water stress. Such information should help provide more insights on the possible mechanism(s) of the enhanced water stress tolerance induced by exogenous Spd during seed germination.

## 2. Results and Discussion 

### 2.1. Effect of Exogenous Spermidine on Seed Germination Characteristics

With the increase of PEG concentration, germination percentage, germination vigor and germination index gradually declined and mean germination time gradually increased in seeds primed with spermidine (Spd) or water, but exogenous Spd apparently improved white clover seed germination and shortened the mean germination time of seeds under water stress (Table 1). Seeds primed with Spd were able to maintain a higher germination percentage, vigor and index than seeds primed with water for the same PEG concentration. When the PEG concentration was 15%, seeds primed with Spd had 8.0%, 8.7% and 19.5% higher germination percentage, vigor and index than seeds primed with water, respectively. Mean germination time in seeds primed with Spd was shortened from 1.11 to 1.04 days and from 1.54 to 1.33 days under 0% and 15% PEG conditions, as compared with seeds primed with water, respectively (Table 1).

**Table 1:** The effect of seed priming with water or spermidine (Spd) on seed germination characteristics in white clover under 7 days of different water stress conditions. Values are mean ± SE (*n* = 6). Different letters in a vertical column indicate a significant difference between each treatment under different PEG concentration. The asterisk indicates a significant difference exists between seed priming with water or Spd. LSD (*p* ≤ 0.05).

PEG (%)	Germination Percentage (%)		Germination Vigor (%)		Germination Index		Mean Germination Time (d)	
	Water	Spd	Water	Spd	Water	Spd	Water	Spd
0	95.5 ± 2.5 a	97.5 ± 1.0 a	95.5 ± 2.5 a	97.5 ± 5.1 a	45.38 ± 1.65 a	47.88 ± 0.48 a *	1.11 ± 0.05 d	1.04 ± 0.03 d *
10	92.0 ± 1.6 a	96.5 ± 1.0 a *	87.3 ± 8.3 a	94.7 ± 2.3 a	39.62 ± 4.15 b	43.56 ± 1.80 b	1.41 ± 0.32 c	1.27 ± 0.12 c
15	78.7 ± 3.1 b	86.7 ± 3.6 b *	74.7 ± 3.1 b	83.3 ± 5.0 b *	31.93 ± 2.20 c	38.16 ± 1.97 c *	1.54 ± 0.11 b c	1.33 ± 0.04 c *
18	54.7 ± 8.1 c	59.5 ± 4.4 c	53.3 ± 4.1 c	58.7 ± 4.2 c	16.53 ± 0.55 d	21.98 ± 1.21 d *	1.72 ± 0.18 b a	1.70 ± 0.11 b
20	23.5 ± 6.6 d	26.0 ± 7.1 d	23.0 ± 7.0 d	25.0 ± 8.2 d	6.36 ± 1.87 e	6.86 ± 1.90 e	1.89 ± 0.10 a	1.90 ± 0.12 a

Under different water stress conditions (0% to 20% PEG), seedling fresh weight in both treatments declined significantly and exogenous Spd significantly increased seedling fresh weight when the PEG concentration was 10% (Table 2). In addition, exogenous Spd treatment apparently simulated growth of roots, and led to a significant increase in seedlings dry weight under water stress. Seedlings dry weight of exogenous Spd treatment increased by about 38% and 19% over non-Spd treatment at 10% and 15% PEG, respectively. A significantly higher seeds vigour index also was observed in seeds priming with Spd than that in seeds priming with water at 15% PEG stress (Table 2).

**Table 2:** The effect of seed priming with water or spermidine (Spd) on fresh weight, dry weight, root length and seed vigour index in seeds of white clover after 7 days of germination under different water stress. Values are mean ± SE (*n* = 6). Different letters in a vertical column indicate a significant difference in each treatment under different PEG concentration conditions. The asterisk indicates a significant difference exists between seed priming with water or Spd. LSD (*p* ≤ 0.05).

PEG (%)	Seedling Fresh Weight (mg·10 Seedling^−1^)		Seedling Dry Weight (mg·10 Seedling^−1^)		Root Length (cm)		Seed Vigour Index	
	Water	Spd	Water	Spd	Water	Spd	Water	Spd
0	86.7 ± 4.8 a	86.1 ± 4.4 a	5.5 ± 0.4 b	5.6 ± 0.07 c	2.97 ± 0.15 b	3.46 ± 0.15 a *	4.02 ± 0.48 a	4.01 ± 0.17 a
10	55.3 ± 6.3 b	65.1 ± 2.4 b *	5.7 ± 0.6 b a	8.0 ± 0.10 a *	3.20 ± 0.03 a	3.45 ± 0.21 a *	2.26 ± 0.45 b	2.84 ± 0.10 b
15	47.4 ± 1.1 c	50.7 ± 3.6 c	6.0 ± 0.06 b a	7.2 ± 0.03 b a *	2.69 ± 0.10 c	2.88 ± 0.12 b	1.52 ± 0.14 c	1.91 ± 0.09 c *
18	37.4 ± 2.9 d	40.5 ± 1.4 d	5.9 ± 0.09 b a	6.5 ± 0.10 b	2.23 ± 0.22 d	2.37 ± 0.08 c	0.70 ± 0.18 d	0.88 ± 0.01 d
20	24.2 ± 4.5 e	24.4 ± 1.2 e	6.2 ± 0.02 a	6.7 ± 0.03 b	1.47 ± 0.05 e	1.60 ± 0.14 d	0.16 ± 0.07 e	0.23 ± 0.02 e

### 2.2. Effect of Exogenous Spermidine on Carbohydrate Levels

**Figure 1:**
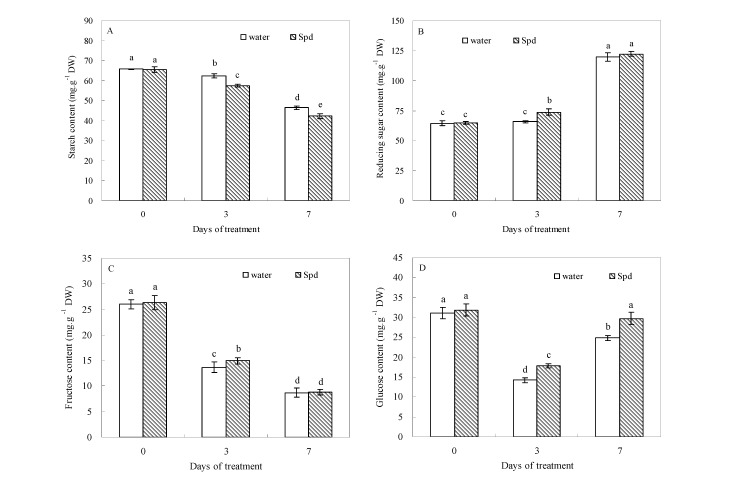
The effect of seed soaking with water or spermidine (Spd) on (**A**) starch content; (**B**) reducing sugar content; (**C**) fructose content and (**D**) glucose content in the process of seeds germination (7 days) under water stress induced by 15% PEG. Vertical bars indicate ± SE of mean (*n* = 6). Different letters above columns indicate significant difference. LSD (*p* ≤ 0.05).

Four organic carbohydrates were detected in the seeds of both treatments. At the beginning of germination (0 day), the contents of starch, reducing sugar, fructose and glucose were not significantly different between the two treatments. Seed starch content decreased, whereas reducing sugar content increased in white clover during the process of germination (Figure 1A,B).

Under water stress, seeds primed with Spd showed further decreased seed starch content at 3 days of germination. In contrast, seeds primed with Spd showed a significantly increased reducing sugar content in germinating seeds. At 3 days of germination, reducing sugar content in exogenous Spd treatment was about 12.2% higher than that in the control (seeds primed with water, Figure 1B). Fructose content began to decline from the beginning to the end of germination in both treatments, but compared to seeds primed with water, exogenous Spd significantly enhanced fructose accumulation after 3 days of germination (Figure 1C). The difference in glucose content was significantly greater in seeds primed with Spd than primed with water under different germination time conditions. Accordingly, Spd treatment resulted in 18.6% and 16.5% higher glucose content than water treatment at 3 and 7 days of germination under water stress (Figure 1D).

### 2.3. Effect of Exogenous Spermidine on Amylase Activities and Gene Relative Expression

**Figure 2:**
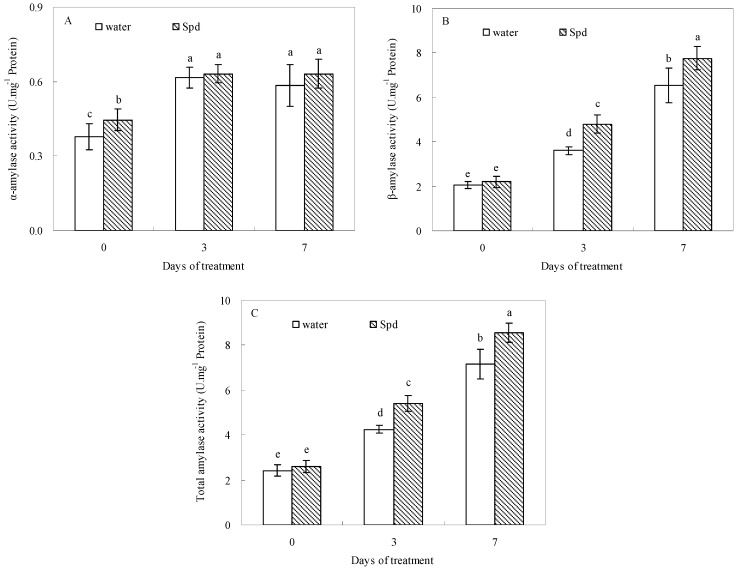
The effect of seed soaking with water or spermidine (Spd) on (**A**) α-amylase activity; (**B**) β-amylase activity and (**C**) total amylase activity in the process of seeds germination (7 days) under water stress induced by 15% PEG. Vertical bars indicate ± SE of mean (*n* = 6). Different letters above columns indicate significant difference. LSD (*p* ≤ 0.05).

Progressive germination induced a significant increase of α-amylase activities, so that α-amylase activities of both treatments reached their maximum after 3 d of water stress. Compared to control (seeds primed with water), exogenous Spd significantly enhanced α-amylase activities at the beginning of the germination progress (Figure 2A). The β-amylase and total amylase activities were also significantly impacted by exogenous Spd, and accordingly a significantly higher β-amylase and total amylase activities in exogenous Spd treatment was observed than that in control at the same level of water stress and germination time (Figure 2B,C).

**Figure 3:**
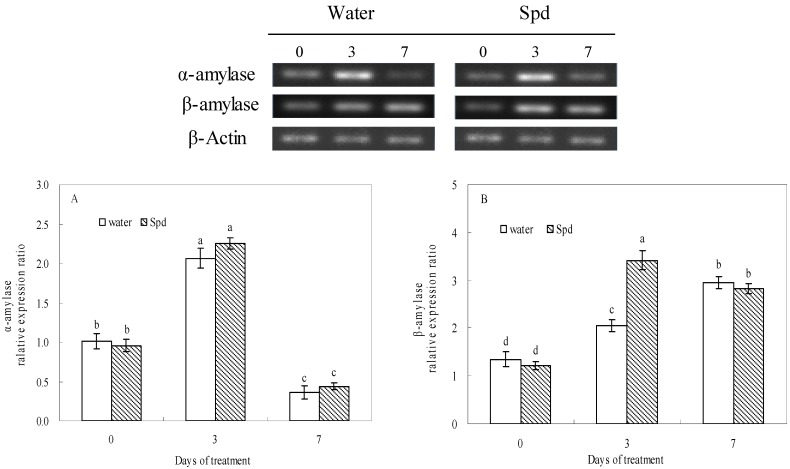
The effect of seed soaking with water or spermidine (Spd) on (**A**) α-amylase gene and (**B**) β-amylase gene relative expression ratio in the process of seeds germination (7 days) under water stress induced by 15% PEG. Vertical bars indicate ± SE of mean (*n* = 4). Different letters above columns indicate significant difference. LSD (*p* ≤ 0.05).

The transcript level of α-amylase gene increased rapidly at 3 days of germinating under water stress a thereafter decreased deeply in both treatments, but there was no significant difference between the two treatments (Figure 3A). The transcript level of β-amylase gene gradually changed with the increase in germination time, and the transcript level of β-amylase gene of exogenous Spd treatment was 1.7 times as high as that of control at 3 d of germination, and the differences were statistically significant (Figure 3B).

### 2.4. Effect of Exogenous Spermidine on Reactive Oxygen Species Production and Membrane Damage

**Figure 4:**
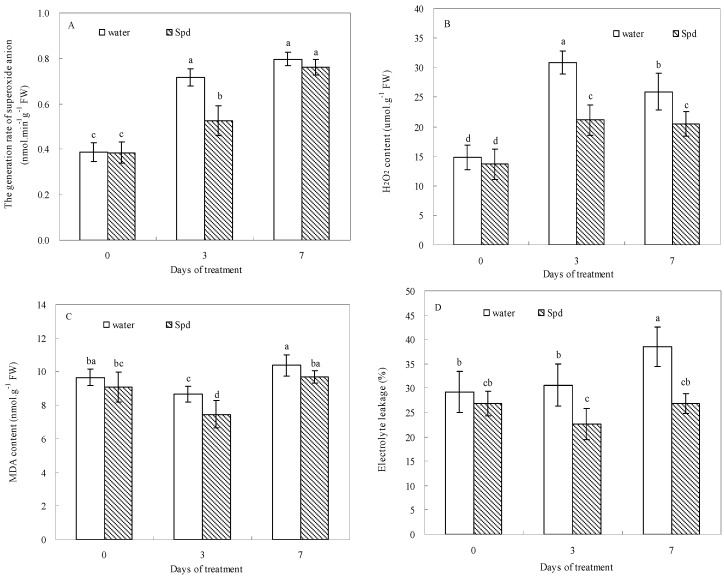
The effect of seed soaking with water or spermidine (Spd) on (**A**) the generation rate of superoxide anion (O_2_^−^); (**B**) H_2_O_2_ content; (**C**) MDA content and (**D**) electrolyte leakage (EL) in the process of seeds germination (7 days) under water stress induced by 15% PEG. Vertical bars indicate ± SE of mean (*n* = 6). Different letters above columns indicate significant difference. LSD (*p* ≤ 0.05).

O_2_^−^ and H_2_O_2_ content of seedlings increased during germination under water stress in both treatments (Figure 4A,B). Exogenous Spd treatment significantly alleviated the water stress-induced accumulation of H_2_O_2_ and MDA and decreased the release rate of O_2_^−^ in the germinating white clover seeds in response to water stress (Figure 4). Electrolyte leakage (EL) level increased with germination time in control, but EL remained relatively stable with Spd treatment and had significantly lower levels relative to control (Figure 4D). Exogenous Spd alleviated water stress-induced membrane damage during germination.

### 2.5. Effect of Exogenous Spermidine on Antioxidant Enzyme Activities and Gene Relative Expression

**Figure 5:**
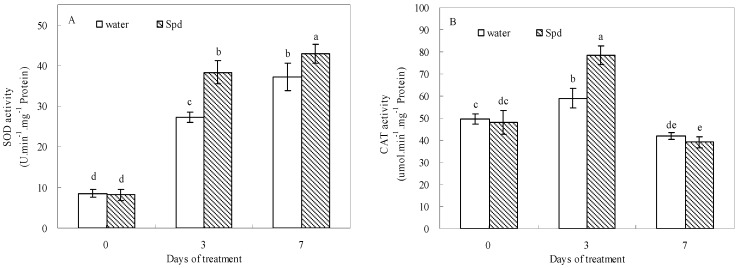

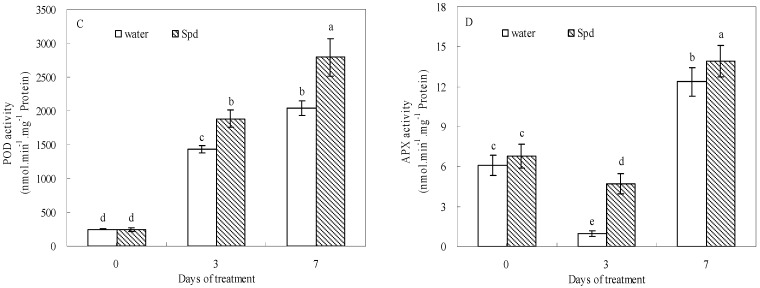
The effect of seed soaking with water or spermidine (Spd) on (**A**) superoxide dismutase (SOD) activity; (**B**) catalase (CAT) activity; (**C**) guaiacol peroxidase (POD) activity and (**D**) ascorbate peroxidase (APX) activity in the process of seeds germination (7 days) under water stress induced by 15% PEG. Vertical bars indicate ± SE of mean (*n* = 6). Different letters above columns indicate significant difference. LSD (*p* ≤ 0.05).

Exogenous Spd had stimulative effects on SOD, POD, CAT and APX activities during germination under water stress conditions (Figure 5). In response to water stress, SOD and POD activities gradually increased and then reached a peak value at the last day of germination in both treatments. Moreover, SOD and POD activities after Spd treatment were significantly higher than those of control at 3 days and 7 days of germination (Figure 5A,C).

At the beginning of germination, CAT activities showed a gradual increase for both treatments and then started to decline following aggravating stress, but CAT activity was elevated significantly with Spd treatment as compared with that in control at 3 d (Figure 5B). The APX activity of Spd treatment also was significantly higher than that of the control, and the difference was most pronounced at 3 d of germination under water stress (Figure 5D).

**Figure 6:**
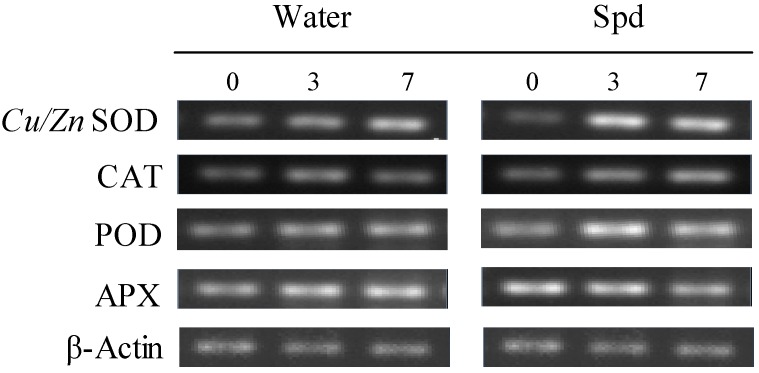

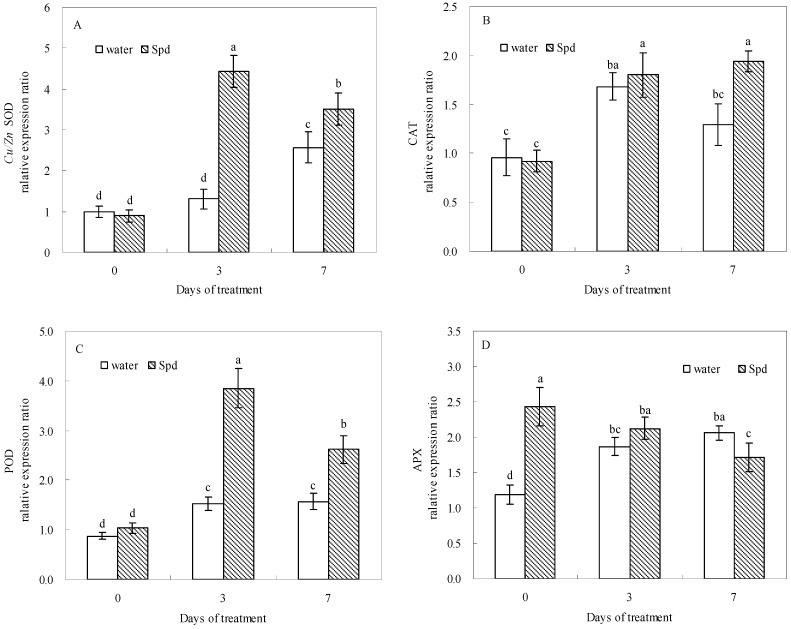
The effect of seed soaking with water or spermidine (Spd) on (**A**) superoxide dismutase (SOD) gene; (**B**) catalase (CAT) gene; (**C**) guaiacol peroxidase (POD) gene and (**D**) ascorbate peroxidase (APX) gene relative expression ratio in the process of seeds germination (7 days) under water stress induced by 15% PEG. Vertical bars indicate ± SE of mean (*n* = 4). Different letters above columns indicate significant difference. LSD (*p* ≤ 0.05).

Under water stress, the exogenous Spd-treated seeds showed higher transcript levels of SOD, CAT, POD and APX genes compared with control during germination (Figure 6). At 0 day of germination, seeds SOD, CAT and POD gene transcript levels in both treatments kept the similar level without statistically significant differences, whereas SOD, POD and CAT gene transcript levels after Spd treatment is 3.4 and 2.5 times at 3 days of germination and 1.5 times at 7 days of germination that of control under water stress, respectively (Figure 6A–C).

A significantly higher APX genes relative expression ratio was observed in seeds priming with Spd compared to that in seeds priming with water after 3 h of soaking (0 day). After that, the transcript level of APX genes began to decline in Spd treatment and increase in control, and showed different expression patterns (Figure 6D).

**Table 3:** The relationship between the enzyme activities and gene expressions of enzymes; α-amylase, β-amylase, Cu/ZnSOD, CAT, POD and APX in the process of seeds germination, submitted to water stress induced by 15% PEG for 0 day, 3 days and 7 days. “+” induced; “—” suppressed; “ns”, not significantly changed as compared with seeds priming with water (control) according to SE at *p* ≤ 0.05.

	Enzyme Activity			Gene Expression		
	0 day	3 days	7 days	0 day	3 days	7 days
α-amylase	+	ns	ns	ns	ns	ns
β-amylase	ns	+	+	ns	+	ns
*Cu/Zn*SOD	ns	+	+	ns	+	+
CAT	ns	+	ns	ns	ns	+
POD	ns	+	+	ns	+	+
APX	ns	+	+	+	ns	—

The relations between gene expressions and enzyme activities were examined (Table 3). Under water stress, a positive correlation between the biochemical and the molecular levels of enzyme-gene expression was found in seedlings of Spd-treated white clover at 3 d of treatment for β-amylase, SOD and POD, whereas a negative correlation was found at 7 d of treatment for APX. For α-amylase and CAT, a parallel induction of genes and enzymatic activities was observed as response to water stress at 3 and 7 d of treatment (Table 4).

**Table 4:** The effect of seed priming with water or spermidine (Spd) on ascorbic acid (AsA) and dehydroascorbic acid (DAsA) content in seeds of white clover after 7 days of germination under 15% PEG. Values are mean ± SE (*n* = 6). Different letters in a vertical column indicate significant difference between two treatments. LSD (*p* ≤ 0.05).

Treatment	Content (umol.g^−1^ FW)			
	AsA	DAsA	AsA + DAsA	AsA/DAsA
Water	18.77 ± 0.66 b	3.45 ± 0.46 a	20.97 ± 1.31 b	5.21 ± 1.14 b
Spd	21.42 ± 1.46 a	1.52 ± 0.20 b	23.59 ± 1.04 a	14.59 ± 1.33 a

### 2.6. Effect of Exogenous Spermidine on Ascorbic Acid and Glutathione Content

Total ascorbic acid, ascorbic acid (AsA), dehydroascorbic acid (DAsA) and the DAsA/AsA concentrations are shown in Table 5. Exogenous Spd improved significantly the ascorbic acid (AsA), total ascorbic acid (AsA + DAsA) and the DAsA/AsA levels in white clover seedlings under water stress. On the contrary, dehydroascorbic acid (DAsA) content decreased by 56% with Spd treatment compared with that in control when exposed to 7 days of germination and showed a statistically significant difference (Table 5). There were significant differences on reduced glutathione (GSH), total glutathione (GSH+GSSG) content and GSH/GSSG between two treatments at 3 days of germination (Table 5). The 43% increase of GSH/GSSG in Spd treatment was observed relative to control and that indicated that the GSH turnover rate remained higher in Spd treatment when seeds germination of white clover under water stress.

**Table 5:** The effect of seed priming with water or spermidine (Spd) on reduced glutathione (GSH) and glutathione disulfide (GSSG) content in seeds of white clover after 7 days of germination under 15% PEG. Values are mean ± SE (*n* = 6). Different letters in a vertical column indicate significant difference between two treatments. LSD (*p* ≤ 0.05).

Treatment	Content (umol.g^−1^ FW)			
	GSH	GSSG	GSH+GSSG	GSH/GSSG
water	1.37 ± 0.423 b	2.01 ± 0.21 a	3.29 ± 0.27 b	0.75 ± 0.21 b
Spd	2.29 ± 0.21 a	1.86 ± 0.11 a	4.01 ± 0.30 a	1.07 ± 0.19 a

### 2.7. Effect of Exogenous Spermidine on Root Viability

**Figure 7:**
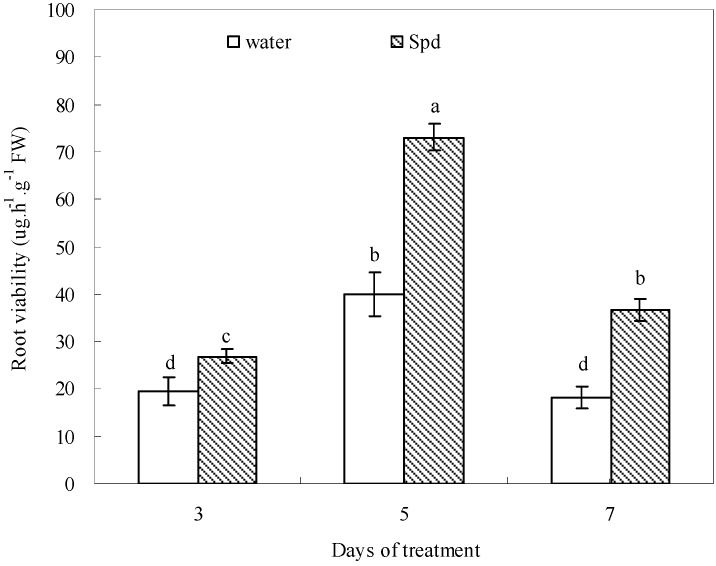
The effect of seed soaking with water or spermidine (Spd) on root viability in the process of seeds germination (7 days) under water stress induced by 15% PEG. Vertical bars indicate ± SE of mean (*n* = 6). Different letters above columns indicate significant difference. LSD (*p* ≤ 0.05).

Root viability was significantly enhanced in seeds priming with Spd than that in seeds primed with water during the germination progress, and the difference was most pronounced at 7 days of germination. The root viability increased in response to 15% PEG to approximately 28%, 83% and 100% of the control (seeds priming with water) in seeds priming with Spd at 3, 5 and 7 days of germination, respectively (Figure 7).

### 2.8. Discussion

Seed germination is a complicated physiological process modulated by phytohormones or physiological activator such as abscisic acid [29], nitric oxide [30] or polyamines [31]. Vigorous seedlings were receptive to effective seed priming with polyamines (PAs), and were evidently better able to resist the adverse effects of drought [25]. Recently, it has been postulated that PAs can improve the cell water status in rice, thereby producing better growth under limited water supply conditions [32]. Although the effect of all the PAs was stimulatory, spermidine (Spd) was statistically effective in most of the attribute studies [18,33]. The results of our study revealed that seeds primed with Spd not only showed improved germination percentages and shortened mean germination times but also displayed significantly enhanced seed vigor as indicated by longer root length, seedling fresh and dry weights compared with control. The result is consistent with an earlier study of Sinska and Lewandowska *et al.* [33] about the effect of exogenous Spd on apple seeds. Farooq *et al.*, [2] found a strong correlation between amylase and soluble sugars which supported the assumption that speedy starch metabolism contributed towards the early emergence of seeds and vigorous seedling growth induced by priming with Spd. Metabolites of starch such as glucose are critical for seed germination as they are involved as osmolytes for cellular turgor maintenance and energy sources. Under water deficit, seed priming with Spd improved starch metabolism as indicated in this study by elevated α- and β-amylase activities in agreement with the results of Farooq *et al.*, [2]. The data revealed that Spd plays an important role in accelerating starch metabolism during seed germination in response to water stress.

One of intricate effects of drought is loss of integrity of biological membranes due to oxidative damage; accordingly, maintenance of balance between the generation and quenching of reactive oxygen species (ROS) in plants is crucial to survive water stress [34,35]. Many previous studies have showed that PAs reduced ROS levels, for instance, under osmotic stress exogenous PAs inhibited the accumulation of O_2_^−^ and H_2_O_2_ in wheat seedlings and barley leaves [36,37]. Transgenic pear exhibited more accumulation of Spd when exposed to osmotic stress and displayed higher SOD and APX activities, less MDA and H_2_O_2_ content than the wild type, implying it suffered from less injury [38,39]; on the contrary, antisense inhibition of a Spd synthase gene reduced the antioxidant system significantly in pear accompanied by a greater accumulation of MDA compared with wild-type. Growth inhibition, Spd level, and antioxidant system were significantly ameliorated by exogenous Spd application in pear shoots [40]. In current study, exogenous Spd significantly promoted activities of SOD, POD, CAT and APX relative to untreated seeds at different stress and germination time. The same result was obtained in study of Li [41] about effect of exogenous Spd on wheat seedlings under osmotic stress. The ascorbate-glutathione cycle (AsA-GSH cycle) is the major non-enzymatic antioxidant process scavenging H_2_O_2_ in different cellular compartments [42]. Significantly higher AsA/DAsA and GSH/GSSG ratios in seeds primed with Spd were observed in this study, which suggested that Spd accelerated the regeneration of AsA and GSH when seeds were germinated under water deficit. As a result, enhanced antioxidant enzyme activities and AsA-GSH cycle in Spd-treated seeds resulted in lower the generation rate of O_2_^−^, H_2_O_2_ and MDA content and improved cell membrane stability, as demonstrated by lower electrolyte leakage (EL). This indicates that Spd is able to influence the antioxidant defense system to moderate the oxidative stress intensity induced by water stress during seed germination. These results further confirm the action of Spd acts as ROS scavengers.

The role of PAs in root mortality and senescence caused by water deficit remains less well-documented. It has been reported that a drought-tolerant white clover cultivar exhibited significantly higher root viability than a drought-sensitive one under drought-stressed conditions [43]. Our results showed that Spd may be involved in regulating root mortality, since seeds primed with exogenous Spd had significantly increased root viability and length during germination under water stress. According to the report of Xu *et al.* [44], root was more easily affected by Put than shoot during seed germination in chilling-stressed tobacco. High Spd level was characteristic of meristematic cells and might be essential for the development of globular structure and root length [45,46]. It is possible that the increase in antioxidant defense and the maintenance of higher starch metabolism in Spd-treated seeds under water stress could have stimulated root antioxidants to reduce root lipid peroxidation to maintain root survival and transported adequate carbon to the roots to maintain root growth. However, the accurate mechanism deserves more investigation, especially the effects of Spd on root tip viability when seed germination was subjected to water deficit.

PAs can regulate many enzyme activities by bonding with the enzyme protein or participation in the process of phosphorylation of the enzyme protein [47,48]. It was suggested that PAs were implicated in directly regulating antioxidant enzyme activities, thereby reducing oxidative stress [49]. Apart from such direct stress protective roles, PAs may also play a role as a signaling molecule in plant responses to stress [28]. Spd and Spm in particular promote gene expression and increase the DNA-binding activity of transcription factors [50,51]. The results from RT-PCR analysis in the present study showed that exogenous spermidine strongly affected the expression of genes encoding antioxidant enzymes and β-amylase during white clover seed germination subjected to water deficit, but there was no significant influence on the transcript level of α-amylase. However, exogenous Spd significantly enhanced α-amylase activity under the same water stress condition and germination time. As far as we know, the α-amylase is synthesized *de novo* in the aleurone layer surrounding the endosperm in the process of seed germination. In contrast, a precursor form of β-amylase is present in seeds in the dry state as a proenzyme devoid of enzyme activity, which is activated during germination after cleavage of a peptide sequence at the C-terminal part of the enzyme [12]. These results suggested that Spd might not only enhance the activities of α-amylase and β-amylase, but also induce the *de novo* β-amylase synthesis by elevating β-amylase gene expression at the early stage of white clover seed germination under water stress. Moreover, the better germination of Spd-treated seeds under water stress could be associated with increased expression of specific genes encoding antioxidant enzymes, which partly influenced or improved antioxidant enzyme activities under stress. For antioxidant enzymes, the gene expression pattern did not always go along with their activities changes. The discrepancy between gene expression and enzyme activity indicates enzyme activity changes were not only caused by mRNA levels, but also regulated at the post-transcriptional level and influenced by cellular metabolism [44,52]. The current research also supports the notion that the function of Spd is involved in both sides as a stress-protecting compound and signaling molecule.

## 3. Experimental Section

### 3.1. Plant Materials and Treatments

Seeds of white clover (*Trifolium repens* cv. Ladino) were used as an experimental material. Seeds were surface-sterilized for 5 min in 0.1% mercuric chloride, and rinsed four times with distilled water. Two treatments were set in this experiment. One set of seeds was soaked in distilled water as control and another set of seeds was soaked in 30 μM spermidine (Spd) for 3 h at 20 °C, respectively. The soaked seeds were then germinated in Petri dishes with a diameter of 9 cm containing two sheets of filter paper moistened initially with 10 mL of 0%, 10%, 15%, 18% and 20% (W/V) Polyethylene glycol 6000 (PEG 6000) and each treatment was replicated six times (100 seeds for each replicate). The Petri dishes were kept at a growth chamber programmed at average day/night temperature of 23/19 °C, 75% relative humidity and 300 μmol∙m^−2^∙s^−1^ photosynthetic photon flux density for 7 days. Different concentrations of PEG 6000 were added every day when necessary. Seeds were sampled at 0 (after soaking in 30 μM Spd or distilled water for 3 h), 3 and 7 days after germination for bio-chemical and physiological measurements, and another batch was sampled at 7 days after germination for determinations of dry weight, fresh weight, root length and seed germination percentage.

### 3.2. Determination of Seed Germination Characteristics

Germination vigor (GV) and germination percentage (GP) were evaluated after 3 and 7 days of germination, respectively. Germination index (GI) and mean germination time (MGT) were calculated based on the following formula:

(1) $$\mathrm{GI}=\sum \frac{\mathrm{Gt}}{\mathrm{Tt}}$$

where *Gt* stands for the number of the germinated seeds in the *t* day; *Tt* stands for time corresponding to *Gt* in days and:

(2) $$\mathrm{MGT}=\frac{\sum \mathrm{Ti}\times \mathrm{Ni}}{\sum \mathrm{Ni}}$$

where *Ni* stands for the number of the new germination seeds in times of *Ti*, respectively [53]. After 7 days of germination, seedlings fresh weight (FW), dry weight (DW), root length (RL) and seed vigour index (VI) were measured, and seedlings dry weight was measured after drying at 105 °C for 2 h, and then maintained at 80 °C for 3 days. Seed vigour index (VI) was determined from seedlings fresh weight and germination index using the formula VI = FW × GI.

### 3.3. Carbohydrate and Amylase Activity

For carbohydrate quantification, the procedure was conducted following the method of Fu and Dernoeden [54]. Seeds (0.5 g) were collected and dried in an oven. Dry tissue (0.05 g) was extracted in 92% ethanol (1 mL) and centrifuged at 20,000 *g* for 10 min. The supernatant was used to measure content of reducing sugars (glucose and fructose), and the residue was obtained for starch content analysis. An aliquot of supernatant (1 mL) was combined with ferricyanide reagent (1.25 mL) and placed in a water bath at 100 °C for 10 min. After cooling tubes to room temperature, 2 N H_2_SO_4_ (2.5 mL) was added followed by arsenomolybdate reagent (1 mL). The absorbance of the solution was measured at 515 nm using a spectrophotometer, and reducing sugar content was calculated based on a glucose standard curve as described by Ting [55]. Starch content was measured using the method described by Smith [56].

The activities of amylase enzymes were measured using the method of Tarrago and Nicolas [57] and Kishorekumar *et al.* [58]. Seeds (0.1 g) were ground with distilled water (8 mL) at 4 °C. The extract was centrifuged at 20,000 *g* for 25 min at 4 °C. The supernatant was used for estimating α-amylase and β-amylase activities. Supernatant (3 mL) and CaCl_2_ (3 mM, 3 mL) were mixed and incubated at 70 °C for 5 min. The reaction mixture (0.1 mM citrate buffer, 2% soluble starch solution, 0.7 mL hot enzyme extract) was incubated at 30 °C for 6 min and then the mixture was heated for 5 min at 50 °C. The α-amylase activity was estimated spectrophotometrically at 540 nm. After inactivating α-amylase at pH 3.4, the β-amylase activity was determined. Reaction solution (2 mL, 0.1 mM citrate buffer, 2% soluble starch, 0.7 mL EDTA treated enzyme extract) was incubated at 30 °C for 5 min after the addition of starch. The β-amylase activity was then assayed as described for α-amylase.

### 3.4. Reactive Oxygen Species and Electrolyte Leakage

The rate of formation of O_2_^−^ was measured using the sulfanilamide method [59] and the absorbance was measured at 530 nm. H_2_O_2_ was assayed by the potassium iodide method. The oxidation product was measured at 390 nm. The amount of H_2_O_2_ formed was computed from the standard curve made earlier with known concentrations of H_2_O_2_ [60].

For electrolyte leakage (EL), samples of fresh seeds (0.1 g) were immersed in the centrifuge tube with deionized water (15 mL). The tubes were shaken for 24 h on a shaker table. The conductivity of the solution (C_initial_) was measured using a conductivity meter (DDS-307A, Shanghai Precision and Scientific Instrument Co., Ltd., Shanghai, China). Seeds then were killed by autoclaving at 140 °C for 30 min. The conductivity of killed tissues (C_max_) was measured. Relative EL was calculated as the percentage of C_initial_ over C_max_ [61].

### 3.5. Antioxidant Enzyme Activity, Ascorbic Acid, Glutathione and Malondialdehyde Content

To analyze the antioxidant enzyme activities, fresh seeds (0.2 g) were randomly sampled from each pot at each sampling date, frozen in liquid nitrogen immediately, and stored at −80 °C until use. For extraction, the frozen sample was ground on ice with 50 mM cold phosphate buffer (4 mL, pH 7.8) containing 1% (w/v) polyvinylpyrrolidone. The homogenate was centrifuged at 12,000 *g* for 30 min at 4 °C. The supernatant was used for assays of antioxidant enzyme activity and content of malondialdehyde (MDA), which was measured as the degree of lipid peroxidation. The SOD activity was measured by recording the rate of *p*-nitroblue tetrazolium chloride reduction of the absorbance at 560 nm [62]. The activity of CAT, POD and APX was determined by following the changes in absorbance at 240, 470 and 290 nm, respectively [63,64]. Protein content was determined using Bradford’s [65] method. Ascorbic acid (AsA), dehydroascorbic acid (DAsA), reduced glutathione (GSH) and glutathione disulfide (GSSG) content were measured using the method of Gossett *et al.* [66].

The content of MDA was measured using the method of Dhindsa *et al.*, [67] with some modification. Enzyme extract (0.5 mL) and reaction solution (1 mL) containing 20% w/v trichloroacetic acid and 0.5% w/v thiobarbituric acid were added to the pellet. The mixture was heated in a water bath at 95 °C for 15 min, and then cooled quickly in an ice-water bath. The homogenate was centrifuged at 8000 *g* for 10 min. The absorbance of the supernatant was measured at 532, 600 and 450 nm. The concentration of MDA was calculated by subtraction of OD_600_ from OD_532_ and OD_450_.

### 3.6. Root Viability

Root viability was determined by the method of Knievel [68] by using the triphenyltetrazolium chloride (TTC) method with some modification. Fresh roots (0.1 g) were randomly sampled from each pot at each sampling date, frozen in liquid nitrogen immediately and until use. The frozen sample, 0.4% TTC (5 mL) and 1/15 M phosphate buffer (5 mL, pH 7.0) were added to the pellet and incubated at 37 °C for 1 h in the dark. Then 1 M sulfuric acid (2 mL) was added. After taking the roots out and putting into a new pellet, methanol (20 mL) was added and incubated at 40 °C for 7 h. The absorbance of the supernatant was measured at 280 nm.

### 3.7. Gene Expression Analysis

All genes used in this study, except POD gene (GenBank ID: AJ011939), β-amylase gene (GenBank ID: AF049098.1) and the reference genes β-actin (GenBank ID: JF 968419), were identified based on sequence similarity after TBLASTX analysis with related genes from white clover expressed sequence tags (ESTs). Primers were manually designed based on the EST sequences. Candidate sequences were further confirmed by sequencing after PCR amplification using the same primers. The white clover homologues of α-amylase, SOD, CAT and APX genes were identified based on sequence similarity with cicer arietinum α-amylase gene (GenBank ID: XM_004514715.1), the red clover SOD gene (GenBank ID: AY434497), broad bean CAT gene (GenBank ID: JQ043348) and alfalfa APX gene (GenBank ID: XM_003601995). The putative white clover α-amylase, SOD, CAT and APX genes displayed 91%, 94%, 93% and 95% identity with cicer arietinum α-amylase gene, red clover SOD gene, broad bean CAT gene and alfalfa APX gene, respectively. Primers used for RT-PCR are presented in Table 6.

**Table 6:** Primer sequences and their corresponding GeneBank accession numbers of the analyzed genes.

Target Gene	Accession No.	Forward Primer (5'–3')	Reverse Primer (5'–3')
α-amylase	FY463398.1	ATGGATGCGACCAAACCTTACT	GCAGCACTAATCCAGTCAACGA
β-amylase	AF049098.1	TCAGCAGGTATTGAGTGGAGGTT	TTGACACCTTGTGGTCTTGCAT
*Cu/Zn*SOD	FY461274	TCACCTTCCACTTTCAAACCTCTC	TGTTGGACCTTCGTCTTCTTGAGT
CAT	FY464988	GTCTTCTTTGTTCACGATGGGATG	GAAAGTGGGAGAAGAAGTCAAGGAT
POD	AJ011939	TCTAGGGCAACGGTTAATTCATTC	GGTACGGATTTTCCCATTTCTTG
APX	FY460674	GCAGCATCAGTTGGCAAGACC	GGCAAACCTGAGACTAAATACACGA
β-Actin	JF968419	TTACAATGAATTGCGTGTTG	AGAGGACAGCCTGAATGG

Gene expression was performed using a reverse transcriptase polymerase chain reaction (RT-PCR). Total RNA was extracted from seeds with RNeasy Mini Kit (Qiagen, Suzhou, China) according to the manufacturer’s protocol. RNA concentration was calculated from the optical density of the samples at 260 nm. RNA was reverse-transcribed with Revert Aid First Stand cDNA Synthesis Kit (Fermentas, Shengzhen, China). The synthesized cDNA was subjected to PCR using primers of α-amylase, β-amylase, SOD, CAT, POD, APX and β-Actin (as internal control). The conditions of the PCR protocol for β-Actin gene, α-amylase, β-amylase, SOD, CAT and POD genes were as follows: 3 min at 94 °C and 30 repeats of denaturation at 94 °C for 30 s, annealing at 57 °C for 30 s and extension at 72 °C for 1 min. And the conditions of the PCR protocol for APX gene was as follows: 3 min at 94 °C and 30 repeats of denaturation at 94 °C for 30 s, annealing at 62.5 °C for 30 s and extension at 72 °C for 1 min. Aliquots of individual PCR products were resolved through agarose gel electrophoresis and images were captured by Quantity One and the bands were also determined with the Discovery Series Quantity One.

### 3.8. Statistical Analysis

The general linear model procedure of SAS 9.1 (SAS Institute, Cary, NC, USA) was used to determine the significance of relationships among the measured variables. Conclusions are based on differences between means significant at *p* ≤ 0.05 by Duncan’s multiple range test.

## 4. Conclusions

In summary, seed priming with Spd can improve the seed germination, root length and viability of white clover under water stress conditions. The physiological effects of exogenous Spd on improving seeds tolerance to water deficit during germination were reflected by lower lipid peroxidation levels, better cell membrane stability and significantly higher seed vigour indexes in white clover seedlings. The maintenance of higher starch metabolism in Spd-treated seeds under water stress may be one of critical factors for seed germination via involvement in osmolytes for cellular turgor maintenance and energy sources, and it also could supply adequate carbon available to transport to the roots to maintain root growth or survival. Enhanced antioxidant enzyme activities, ASC-GSH cycle and expression of genes encoding antioxidant enzymes induced by exogenous Spd offer other possibilities to be involved in acquiring drought tolerance through scavenging ROS in water-stressed white clover seeds. In addition, Spd regulated other metabolic processes involved in seed germination and tolerance under water deficit conditions that may deserve further investigation.
